# Determination of the Phenolic Profile, and Evaluation of Biological Activities of Hydroethanolic Extract from Aerial Parts of *Origanum compactum* from Morocco

**DOI:** 10.3390/molecules27165189

**Published:** 2022-08-15

**Authors:** Mounia Chroho, Aziz Bouymajane, Mustapha Aazza, Yassine Oulad El Majdoub, Francesco Cacciola, Luigi Mondello, Touriya Zair, Latifa Bouissane

**Affiliations:** 1 Molecular Chemistry, Materials and Catalysis Laboratory, Faculty of Sciences and Technologies, Sultan Moulay Slimane University, Beni-Mellal 23000, Morocco; 2 Team of Microbiology and Health, Laboratory of Chemistry-Biology Applied to the Environment, Faculty of Sciences, Moulay Ismail University, Zitoune, Meknes 11201, Morocco; 3 Laboratory of Chemistry-Biology Applied to the Environment, Faculty of Sciences, Moulay Ismail University, Zitoune, Meknes 11201, Morocco; 4 Department of Chemical, Biological, Pharmaceutical and Environmental Sciences, University of Messina, 98168 Messina, Italy; 5 Department of Biomedical, Dental, Morphological and Functional Imaging Sciences, University of Messina, 98125 Messina, Italy; 6 Chromaleont s.r.l., c/o Department of Chemical, Biological, Pharmaceutical and Environmental Sciences, University of Messina, 98168 Messina, Italy; 7 Department of Sciences and Technologies for Human and Environment, University Campus Bio-Medico of Rome, 00128 Rome, Italy; 8 Research Team Chemistry of Bioactive Molecules and Environment, Laboratory of Innovative Materials and Biotechnologies of Natural Resources, Faculty of Sciences, Moulay Ismail University, Zitoune, Meknes 11201, Morocco

**Keywords:** *Origanum compactum*, phenolic compounds, HPLC-PDA-ESI/MS, antioxidant activity, antibacterial activity

## Abstract

*Origanum compactum* belonging to the family Lamiaceae is widely used in food and pharmaceutical fields due to its biologically active substances. We aimed to investigate the total phenol and flavonoid contents and the phenolic composition, and to evaluate the antioxidant and antibacterial properties of hydroethanolic extract from of *Origanum compactum*. Total phenol and flavonoid contents were evaluated using gallic acid and quercetin as standards, respectively, and the phenolic profile was characterized using high-performance liquid chromatography coupled to a photodiode array and electrospray ionization mass spectrometry (HPLC-PDA-ESI/MS). The antioxidant activity was determined by two methods: ferric reducing power (FRAP) assay and the phosphomolybdate method. The antibacterial effect was evaluated against four bacteria (*Escherichia coli*, *Salmonella typhimurium*, *Staphylococcus aureus* and *Listeria monocytogenes*) using the broth microdilution method. The findings show that the total phenolic and flavonoid contents were 107.789 ± 5.39 mg GAE/g dm and 14,977 ± 0.79 mg QE/g dm, respectively. A total of sixteen phenolic compounds belonging to phenolic acids and flavonoids were detected. Furthermore, the extract showed strong antioxidant activity, and displayed a bacteriostatic effect against *Escherichia coli* and *Salmonella typhimuriumn*, and a bactericidal effect against *Staphylococcus aureus* and *Listeria monocytogenes*. Therefore, this study reveals that *Origanum compactum* extracts display potential as antibacterial and natural antioxidant agents for fighting against pathogenic bacteria and preventing oxidative stress.

## 1. Introduction

The *genus Origanum* belonging to the family Lamiaceae (tribe Mentheae) includes 42 species and 18 hybrids found throughout North Africa and Eurasia [1]. In Morocco, the *genus Origanum* is represented by five taxa, namely, *O. elongatum*, *O. grosii*, *O. frontqueri* and *O. vulgare* and *O. compactum*, the most commonly used taxa of *Origanum* [2].

*Origanum compactum* is an endemic plant of Morocco, widely used for its therapeutic and culinary properties. In Moroccan traditional medicine, it is frequently employed in the form of infusions and decoctions to treat a variety of infections (gastrointestinal disorders, gastric acidity, and bronchopulmonary ailments). In traditional Moroccan cuisine, it is the first aromatic ingredient chosen for flavoring some traditional dishes due to its pleasant flavor and spicy fragrance [2,3]. *Origanum compactum’s* vernacular name is “Zaâtar” and is widespread in Morocco. It is reported in the Middle Atlas, Rif and Northern regions [2].

*Origanum compactum* is a member of the compactum section where successive verticillasters are reconciled fake ears contracted terminal, short and globular. The principal morphological characteristic is that it secretes essential oils with a unique flavor due to the presence of secretary organs (glandular and non-glandular trichomes) [4].

*Origanum* species have been investigated for their secondary metabolites, such as phenolic compounds. *Origanum* extracts have attracted more attention recently, and investigations of the total phenolic contents, phenolic profiles and their relation to biological activities have been conducted. The main types of phenolic compounds found in *Origanum* extracts are phenolic acids and flavonoids. Such molecules are compounds with a variety of structures, characterized by having at least one aromatic ring linked with one or more hydroxyl groups. Phenolic compounds are important due to their various physiological functions that help plants adjust to environmental changes and survive (UV protection, disease resistance, pigmentation and growth regulation) [5].

The phytochemical content of *Origanum* species is responsible for its benefits for human health; in vitro and in vivo assays have proved a wide range of pharmacological properties (antioxidant, antibacterial, anti-inflammatory, anti-cancer, antifungal, antiviral, antileishmanial, anti-asthmatic, anti-ulcer, anti-diabetic, and decreased risk of cardiovascular diseases) [3,5].

Despite the numerous studies that have highlighted the phenolic profile and antioxidant and antibacterial powers of *Origanum* extracts, as far as we know, studies of the ethanolic extract of *Origanum compactum* are few or nonexistent. Thus, in the present study, we aimed to investigate the phenolic compounds and the antioxidant and antibacterial activities of hydroethanolic extract from aerial parts of *Origanum compactum* from the Middle Atlas of Morocco (Khenifra).

## 2. Results and Discussion

### 2.1. Phytochemical Screening

Phytochemical screening indicated the presence of secondary metabolites families in *Origanum compactum* aerial parts: alkaloids, catechic tannins, sterols and triterpenes, flavonoids, saponosides, leucoanthocyanins, oses and holosides, and mucilages. On the other hand, gallic tannins and reducing compounds were almost absent.

Gallic acids, tannins, anthocyanes and flavonoids were previously reported as constituents of *Origanum compactum* [6]. Other species belonging to the same genus, e.g., *Origanum vulgare*, have also been proven to contain tannins, mucilages, proteins, alkaloids, steroids, flavanoids, starch and anthraquinones [7].

### 2.2. Polyphenols Extractions Yield

The extraction yield obtained using hydroethanolic solvent at 70% for *Origanum compactum* was 30.60%. A similar yield of polyphenol extraction by methanol from *Origanum compactum* was obtained by Zeroual et al. (31.70%), while the lowest yield (10.30%) was obtained for n-hexane extraction [3]. It has been reported that ethanol and methanol present similar yields in most cases [8].

The yield of extraction depends on the solvent used. A solvent’s effectiveness is mostly determined by its capacity to dissolve particular phenolic groups [8]. Ethanol is one of the solvents that have been used for the extraction of polyphenols from different plants, whether as an aqueous mixture or as absolute ethanol [9], as it provides better results, being also safer for human health [10].

### 2.3. Total Polyphenols and Flavonoids Contents in Origanum compactum Extract

Total phenolic content for the ethanolic extract of *Origanum compactum* was 107.79 ± 5.39 mg GAE/g dm and flavonoid content was 14.98 ± 0.79 mg QE/g dm (Table 1). The total phenolic content recorded was good compared to the total phenolic content in other *Origanum* extracts. The hydro-methanolic extract of *Origanum vulgare* presented total phenolic content of 79–147 mg GAE/g DW [11]. Bower et al. cited total phenolic content of 430 µg of GAE/mg dm for methanolic extract of *Origanum vulgare* leaves [12]. A relatively low total phenolic content (38 mg GAE/200 mL) was reported for the infusion of the leaves and flowers from *Origanum microphyllum* [13].

### 2.4. HPLC-PDA/ESI-MS Analysis

**Figure 1:**
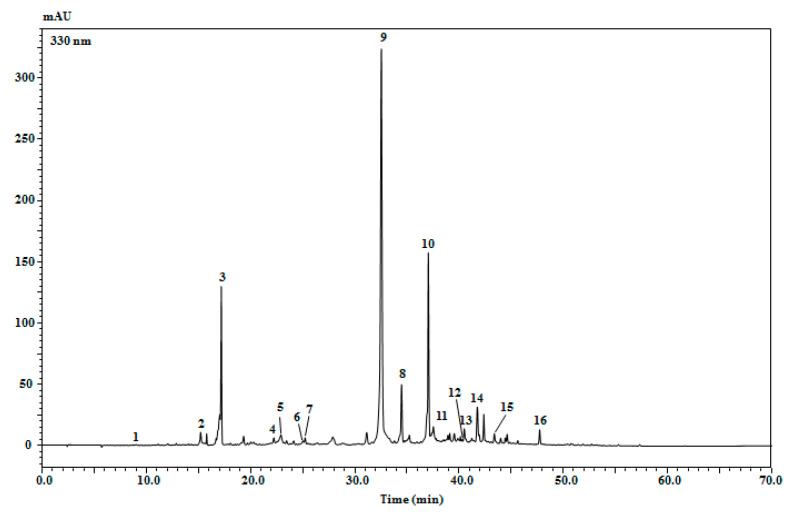
Chromatographic profile of phenolic compounds in *Origanum compactum* extract (EtOH:H_2_O 7:3 *v*/*v*) acquired at 330 nm.

The phenolic profile analysis was carried out by using high-performance liquid chromatography coupled to a photodiode array and electrospray ionization mass spectrometry (HPLC-PDA/ESI-MS) (Figure 1). As listed in Table 2, a total of sixteen phenolic compounds were detected in *Origanum* extract, according to standards, retention times, mass spectrometry and literature data. The compounds were assigned to phenolic acids (syringic acid, caffeic acid, lithospermic acid A isomer, salvianolic acid, rosmarinic acid and melitric acid) and to flavonoids (apigenin-6,8-di-C-glucoside, luteolin glucoside, luteolin glucuronide, diosmetin jaceosidin, apigenin and cirsilineol). In terms of quantification, peak No. 9, rosmarinic acid, turned out to be the most abundant one in the studied plant extract (48,128.62 mg/Kg extract).

As it has been already reported in previous studies, the primary classes of phenolic chemicals in oregano are phenolic acids and flavonoids [5]. The results achieved in this study are in agreement with such studies [5]. For phenolic acids, the majority of them were previously cited in *Origanum* composition. Syringic acid and caffeic acid were previously reported in plants of the Lamiaceae family, including *Oregano* [14], and caffeic acid plays an important role in the biochemistry of this family [23]. Lithospermic acid and caffeic acid were identified in 80% methanol extract of *Origanum vulgare* ssp. *Hirtum* [24]. Lithospermic acid A and B were isolated from the aerial parts of *Origanum. Vulgare* ssp. *Hirtum* by Koukoulitsa et al. [23,25,26]. Salvianolic acid was reported in *Origanum majorana* methanol extract [19], and several studies have reported rosmarinic acid in the *Origanum* phenolic composition [15,20,23,27,28,29]. Additionally, the obtained results showing the high presence of rosmarinic acid in *Origanum compactum* extract are similar to those reported by Boutahiri et al. for the same plant originating from another site in Morocco [29].

It has been reported that rosmarinic acid and derivatives appear to constitute the main phenolic acids in oregano [24]. This is applicable in our study. Rosmarinic acid derivatives combine one or more rosmarinic acids with additional aromatic groups, which include lithospermic acid, salvianolic acid and melitric acid [30].

Regarding melitric acid, it was cited in the phenolic profile of some plants such as *Satureja biflora* [31] and *Melissa officinalis* [30,32], and to our knowledge, no previous studies have reported it as an *Origanum* phenolic component.

Concerning flavonoids, apigenin and lutelin are among the most abundant individual flavonoids found in different extracts of oregano species [5,33]. Cirsilineol was identified in *sicilian oregano* from Italy by Tuttolomondo et al. [34], and diosmetin was cited in flavonoid components of *Origanum vulgare* [35,36]. Jaceosidin was cited in relation to other plants’ phenolic composition; it was isolated from the ethanolic extract of *Centaurea nicaeensis* [37] and was identified as a major phenolic compound in *Artemisia argyi* [38].

### 2.5. Antioxidant Activity

#### 2.5.1. Antioxidant Activity of Hydro-Ethanolic Fractions by Frap (Ferric Reducing Power Assay)

**Figure 2:**
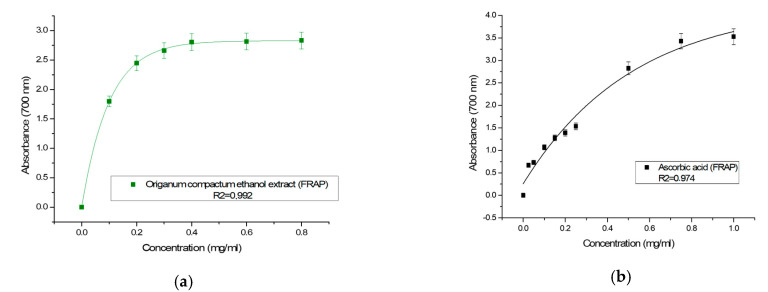
(**a**) Antioxidant activity of hydroethanolic extract of *Origanum compactum* by FRAP; (**b**) antioxidant activity of ascorbic acid by FRAP.

Ethanolic extract of *Origanum compactum* aerial parts (Figure 2) showed powerful antioxidant potential. The parameter EC_50_ (effective concentration), which corresponds to an absorbance equal to 0.5, was equal to 0.017 ± 0.00085 mg/mL (Table 1). For ascorbic acid tested under the same conditions, the EC_50_ was equal to 0.031 mg/mL. The antioxidant power of *Origanum compactum* extract was more powerful than ascorbic acid.

Lagouri et al. compared the ferric reducing antioxidant properties of the aqueous and methanol extracts of *Origanum dictamnus*; the obtained EC_50_ values were 0.028 and 0.038 mg/mL, respectively [39]. In another study, methanolic extract of *Origanum vulgare* showed the largest ferric reducing ability expressed by 1746.76 ± 45.11 µmol AAE/g extract [40].

The powerful ferric reducing power of *Origanum compactum* ethanol extract highlighted in this work is attributed to the phenolic contents of the extract, which are mainly represented by rosmarinic acid and its derivatives. It has been found that rosmarinic acid is the phenolic acid that provides the strongest antioxidant activity by ferric reducing power (FRAP) assay on hydromethanolic extract from *Origanum majorana* [41]. Similarly, Gonçalves et al. [10] partially attributed the high antioxidant capacity (FRAP) of the methanolic extract of *Origanum vulgare* to the large quantity of rosmarinic acid (23.53 mg/g of dry extract) [40].

#### 2.5.2. Total Antioxidant Capacity by Phosphomolybdate Method

The phosphomolybdate method is based on the ability of the extract to convert molybdenum molybdate ions MoO_4_^2−^ into molybdenum molybdate ions MoO_4_^2+^, and the consequent creation of a green phosphate/molybdenum (V) complex at acidic pH. The amount of ascorbic acid equivalents in one gram of dry extract (mg EAA/1 gE) was used to express the total antioxidant capability of the examined extract. 

Total antioxidant capacity of *Origanum compactum* ethanol extract was found to be 470.905 mg EAA/1 gE (Table 1). According to a different study, the stem’s methanolic extract and leaves’ methanolic extract of Cuban oregano (*Plectranthus amboinicus*) showed important antioxidant potential, with TAC values of 634 µM AAE/g of extract and 782.56 µM AAE/g of extract, respectively [42]. Koldaş et al. noted that Kolda the antioxidant activities of water and methanol extracts of *Origanum vulgare* L. ssp. *Viride* were higher than those of ethyl acetate and hexane extracts in terms of phosphomolybdenum reduction potential [43]. Indeed, it has been reported that the antioxidant power of different *Origanum* extracts depends on the solvents used during their extraction, which has been correlated with the phenolic yield during the process [5].

The biological power of plant extracts is related to their content of polyphenols and flavonoids. Rosmarinic acid and its derivatives salvianolic acid and melitric acid are the main phenolic acids in *Origanum compactum* ethanol extract, while the main flavonoid is apigenin glucoside. The biological effects of rosmarinic acid and its derivatives, which include antioxidant, antibacterial, anti-inflammatory and anti-tumor actions, have been recently highlighted [30,44]. The antioxidant effect of rosmarinic acid is linked to its ability to scavenge free radicals, which increases membrane stability and protection against oxidative damage [45].

### 2.6. Antibacterial Activity

The antibacterial effectiveness of *Origanum compactum* hydroethanolic extract was tested against two Gram-negative bacteria (*Escherichia coli* and *Salmonella typhimirium*) and two Gram-positive bacteria (*Staphyloccocus aureus* and *Listeria monocytogenes*) through a broth microdilution assay. Tested concentrations for MIC and MBC were 166.66, 83.33, 41.66, 20.83, 10.41, 5.20, 2.60, 1.30, 0.65 and 0.32 mg/mL. Table 3 summarizes the results. The MIC values for *Origanum compactum* hydro-ethanolic extract against all tested bacteria ranged from 1.30 ± 0.11 to 41.66 ± 0.19 mg/mL. The extract displayed a bacteriostatic activity against *Escherichia coli* and *Salmonella typhimirium* with MBC/MIC values of 32 and 8, respectively, and a bactericidal activity against and *Staphyloccocus aureus* and *Listeria monocytogenes* with MBC/MIC values of 4 and 2, respectively.

The ethanolic extract of *Origanum compactum* was previously tested against *Escherichia coli* and *Staphyloccocus aureus*, and both strains were sensible to the extract [3].

The resistance of *Escherichia coli* and *Salmonella typhimirium* is related to the fact that Gram-negative bacteria are more resistant than Gram-positive bacteria to the majority of antibacterial agents. The resistance of Gram-negative bacteria is attributed to the hydrophilic nature of the membrane, which prevents the passage of hydrophobic molecules such as polyphenols. *Escherichia coli* have a bacterial wall where lipopolysaccharides are abundant and inhibit hydrophobic molecules from passing through the membrane [3].

The antibacterial effect of the ethanolic extract of *Origanum compactum* could be attributed to its phenolic composition. Indeed, the antibacterial activity of rosmarinic acid has been studied and proved against several bacterial strains. Even so, the molecular mechanisms and pathways explaining its biological activities have not been thoroughly investigated [30].

## 3. Materials and Methods

### 3.1. Plant Material

The plant was harvested in the region of Khenifra (El Hammam) in late June 2019. It was cultivated in 2009 by the cooperative El Hammam for the valuation of Medicinal and Aromatic Plants, and it was not chemically treated. The site is located in the Middle Atlas of Morocco at a height of 1182 m. Identification of the species was confirmed at the Scientific Institute of Rabat. The collected samples were dried for ten days at room temperature in the shade.

### 3.2. Phytochemical Screening

Phytochemical screening is a qualitative study based on coloring and/or precipitation reactions from different plant extracts. It aims to highlight the important families of secondary metabolites contained in the plant. These extracts were obtained by decoction, infusion or maceration by solvents. Phytochemical screening is also based on the use of several reagents. Table 4 contains the reagents and names of reactions used to recognize different groups.

### 3.3. Extraction of Polyphenols

Extraction of polyphenols from aerial parts of *Origanum compactum* was performed by Soxhlet apparatus using an aqueous ethanolic mixture (70:30, *v*/*v*). First, 30 g of the plant powder was placed in a filter paper cartridge and introduced into the Soxhlet. The flask was filled with 350 mL of the hydroethanolic solvent and extracted several times until the plant was exhausted and the coloring disappeared. Afterwards, the solvent was evaporated and the extract was recovered with warm distilled water.

The yield of the crude extract was calculated in relation to $m_{0}$, the mass of the dry matter at which the solid-liquid extraction was carried out.

$$R\%=\frac{\mathrm{Mass}\;\mathrm{of}\;\mathrm{crude}\;\mathrm{extract}}{\mathrm{Mass}\;\mathrm{of}\;\mathrm{dry}\;\mathrm{matter}\;\mathrm{powder}}\times 100=\frac{m_{0}}{30}\times 100$$

#### 3.3.1. Determination of Total Polyphenolic Contents in *Origanum compactum*

The total phenol content of the extract was determined by the Folin-Ciocalteu method [46]. In a 100 mL volumetric flask, 5 μL of the extract was mixed with 1.5 mL of Folin-Ciocalteu reagent (10%) and 1.5 mL of sodium carbonate (Na_2_CO_3_) at 7.5% (*m*/*v*). Then, the flask was filled with distilled water. The solution was left for 30 min at room temperature. The absorbance was measured at 760 nm

Gallic acid was used as a positive control. The polyphenol content of the studied extract is calculated from the regression equation of gallic acid calibration (y=0.095x+0.003). The results are expressed in milligrams of gallic acid equivalent per gram of dry matter (mg GAE/g dm). The polyphenol content is calculated using the following formula:

$$T=\frac{C\times V}{m\;\left(\mathrm{dry}\;\mathrm{matter}\right)}\times D$$

where C is the concentration measured by the regression equation of gallic acid calibration, V is the sample volume and D is the dilution factor.

#### 3.3.2. Determination of Flavonoids Contents in *Origanum compactum*

Flavonoid content was estimated by the aluminum trichloride (AlCl_3_) method [47]. First, 10 μL of each fraction was mixed with 0.1 ml of aluminum trichloride 10%, followed by 20 mL of distilled water and supplemented at 50 mL with absolute methanol. The solution was incubated in darkness at room temperature for two hours, and the absorbance was measured at 430 nm. Flavonoids were quantified using a calibration curve performed with the quercetin standard (y=0.073x−0.081). The flavonoid content is expressed in milligrams of quercetin equivalent per gram of dry matter (mg QE/g dm).

### 3.4. HPLC-PDA-ESI/MS Analysis

The ethanolic extract of *Origanum compactum* was analyzed by high-performance liquid chromatography coupled to a photodiode array and electrospray ionization mass spectrometry (HPLC-PDA-ESI/MS). Identification of compounds was performed by using standard compounds (target identification) or by comparing mass spectrum obtained with literature data (tentative identification).

#### 3.4.1. Sample Preparation

The crude ethanolic extract of *Origanum compactum* was redissolved in the same organic solvents and diluted 1:40 (*v*/*v*). For the chromatographic separation, an injection volume of 5 µL was employed, and the analysis was performed in triplicate.

#### 3.4.2. HPLC-MS Analysis Condition

Chromatographic analysis was accomplished by means of a Shimadzu HPLC system (Kyoto, Japan) equipped with a CBM-20A controller, two LC-20AD dual-plunger parallel-flow pumps, a DGU20A5R degasser, a CTO-20AC column oven, a SIL-30AC autosampler, an SPD-M20A photodiode array detector and an LCMS-2020 single quadrupole mass spectrometer, with the employment of ESI source operated in negative and positive ionization modes.

Chromatographic separations were carried out on Ascentis Express RP C18 columns (150 × 4.6 mm; 2.7 μm) (Merck Life Science, Merck KGaA, Darmstadt, Germany). The employed mobile phase was composed of two solvents, water (solvent A) and ACN (solvent B), both acidified with 0.10% of formic acid *v*/*v*. The flow rate was set to 0.8 mL/min, and was split into 0.2 mL/min prior to ESI-MS detection, under gradient elution 0–15 min, 0–15% B; 30 min, 20% B; 60 min, 50% B; 70 min, 100% B; 79 min, 100% B. The injection volume was 5 μL. Diode array detection (DAD) was applied in the range of 200–400 nm and monitored at a wavelength of 330 nm (sampling frequency: 40.0 Hz, time constant: 0.08 s). MS conditions were as follows: scan range and scan speed were set to a mass-to-charge ratio (*m*/*z*) of 100–1000 and 2500 amu/s, respectively; event time was 0.3 s; nebulizing gas (N2) flow rate was 1.5 L/min; drying gas (N_2_) flow rate was 15 L/min; interface temperature was 350 °C; heat block temperature was 300 °C; desolvation line temperature was 300 °C; desolvation line voltage was 1 V; interface voltage was −4.5 kV.

#### 3.4.3. Standards Employed

Calibration curves of three polyphenolic standards (kaempferol-3-glucoside, apigenin, rosmarinic acid) were employed for the quantification of the polyphenolic content in sample extracts. Each analysis was performed in 6 repetitions. Data acquisition was performed by Shimadzu LabSolution software ver. 5.99. Kaempferol-3-glucoside (1, 10, 20, 50, 150) ppm; y = 13848x + 2354.1; R² = 0.9995; LoD = 0.090; LoQ = 0.274. Apigenin (1, 10, 25, 50, 100) ppm; y = 25625x + 608.11; R² = 0.9997; LoD = 0.0263; LoQ = 0.0798. Rosmarinic acid (5, 10, 25, 50, 100) ppm; y = 7150.6x + 9821.3; R² = 0.9993; LoD = 0.14; LoQ = 0.43.

### 3.5. Antioxidant Activity

The antioxidant activity was estimated by two methods: ferric reducing power (FRAP) assay and the phosphomolybdate method (total antioxidant capacity, TAC).

#### 3.5.1. Ferric Reducing Power (FRAP) Assay

Ferric reducing power assay is a simple and inexpensive procedure that estimates the antioxidant level of a sample. It is based on the reducing potential of antioxidants in the extract that react with ferric ions (Fe^3+^) provided by potassium ferricyanide K_3_Fe(CN)_6_ and reduce them to ferrous ions (Fe^2+^). The method used is that described by Zovko Koncic [48].

Extract dilutions with concentrations ranging from 0 to 5 mg/mL were prepared. First, 0.5 mL of each solution was mixed with 2.5 mL of a phosphate buffer solution (0.2 M, pH 6.6) and 2.5 mL of a potassium ferricyanide solution K_3_Fe(CN)_6_ (1%). The mixtures were incubated in a water bath at 50 °C for 20 min. Afterwards, 2.5 mL of trichloroacetic acid (10%) was added to stop the reaction. The mixtures were then centrifuged at 3000 turns for 10 min. At the end, 2.5 mL of the supernatant of each concentration was mixed with 2.5 mL of distilled water and 0.5 mL of an aqueous solution of FeCl_3_ at 0.1%. The absorbance was measured at 700 nm. The increase in absorbance in the reaction medium indicates the increase in the reducing power of the sample. Ascorbic acid was used as a positive control, and its absorbance was measured under the same conditions as the sample.

The antioxidant capacity was expressed by the determination of the effective concentration (EC_50_), which corresponds to an absorbance equal to 0.5. This parameter was used to compare the reducing activity of the sample and the control.

#### 3.5.2. Phosphomolybdate Method (Total Antioxidant Capacity, TAC)

The total antioxidant capacity (TAC) of the ethanolic extract from *Origanum compactum* aerial parts was assessed using the phosphomolybdate method according to Prieto et al., 1999 [49]. This method is based on the use of the plant extract to reduce molybdenum (VI) into molybdenum (V) in an acidic medium. To tubes containing 10 μL of the plant extract with different concentrations, 1 mL of the phosphomolybdate reagent (0.6 M sulfuric acid, 28 mM sodium phosphate and 4 mM ammonium molybdate) was added. After resting at room temperature for 20 min, the tubes were incubated for 90 min at 95 °C. At 695 nm, the absorbance was measured. The data are given as milligrams of ascorbic acid equivalent per gram of extract (mg EAA/g E).

The total antioxidant capacity (TAC) concentration of the analyzed extract was determined using the ascorbic acid calibration curve (y = 0.0411x + 0.0159. R² = 0.9966) and the results are expressed in milligrams of ascorbic acid equivalent per gram of dry extract (mg EAA/1 g E).

### 3.6. Antibacterial Activity

#### 3.6.1. Bacterial Strains and Growth Conditions

The bacterial strains (*Escherichia coli*, *Salmonella typhimirium*, *Staphyloccocus aureus* and *Listeria monocytogenes*) used in this study were obtained from the Laboratory of Microbiology and Health, Faculty of Sciences at Moulay Ismail University of Morocco. Bacterial strains from the frozen stock (−80 °C) were spread on Mueller Hinton agar (Merck Life Science, Merck KGaA, Darmstadt, Germany) and incubated at 37 °C for 24 h. Then, bacterial suspensions were prepared in sterile distilled water and adjusted to the equivalent of 0.5 McFarland standard (108 cfu/mL).

#### 3.6.2. Broth Microdilution Method 

Minimum inhibitory concentration (MIC) and minimum bactericidal concentration (MBC) of extract against four bacterial strains were determined by the broth microdilution method as described by Bouymajane et al. [21]. To sterile, flat-bottom 96-well microplates, 50 µL of Mueller Hinton broth and dimethyl sulfoxide (MHB-DMSO) was added. Then, 50 µL of dried extract mixed with DMSO (500 mg/mL) of *Origanum compactum* was added to the first microplate and mixed in order to determine cascade dilutions. Then, 50 µL of bacterial suspensions and 50 µL of MHB-DMSO were added to each well. The well containing the mixture of bacterial suspensions and MHB-DMSO served as a control, and the well containing the mixture of extract and MHB-DMSO was used as a blank. All microplates were incubated at 37 °C for 24 h. Afterward, 50 μL of TTC (2, 3, 5-triphenyl tetrazolium chloride) was added to each well of the microplates and re-incubated at 37 °C for 30 min. The MIC was determined as the lowest concentration of the extract that showed no visible bacterial growth. The MBC was determined as the lowest concentration of extract that did not produce any bacterial colony. The wells that showed no visible bacterial growth were streaked on Petri dishes containing MHA and incubated 37 °C for 30 min. The MBC/MIC ratio was used to determine the bacteriostatic and bactericidal effects of the extract. If MBC/MIC ≤ 4, the extract effect is bactericidal, and if MBC/MIC > 4, the extract effect is bacteriostatic. All the experiments were carried out in triplicate.

### 3.7. Statistical Analysis

The results are expressed as means ± SD. Statistical analysis was performed by one-way analysis of variance (*ANOVA*) using the SPSS package. All experiments were performed in triplicate and the differences were considered significant at *p* < 0.05.

## 4. Conclusions

We aimed to characterize the phenolic composition and evaluate the antioxidant and antibacterial activities of the hydroethanolic extract from aerial parts of *Origanum compactum*, collected in Morocco. The obtained results reveal the richness of the hydroethanolic extract of *Origanum compactum* in flavonoids and phenolic acids. Furthermore, this extract showed a strong antioxidant capacity and an antibacterial effect, probably due to the presence of luteolin, apigenin and their derivatives, rosmarinic acid and diosmetin. Therefore, based on the obtained results, the aerial parts of *Origanum compactum* are promising as a source of natural antibacterial and antioxidant agents that can be used in food and pharmaceutical fields.

## Figures and Tables

**Table 1:** Total phenols content, flavonoids content and antioxidant activity of hydroethanolic extract from *Origanum compactum*.

Extraction Yield	Total Phenols Content	Flavonoids Content	EC_50_ (FRAP)	TAC
30.60%	107.79 ± 5.39 mg GAE/g dm	14.98 ± 0.79 mg QE/g dm	0.017 ± 0.00085 mg/mL	470.90 mg EAA/g E

**Table 2:** Tentative characterization of phenolic compounds in (EtOH:H_2_O 7:3 *v*/*v*) extract of *Origanum compactum* by HPLC-PDA/MS.

Peak N°	Compound	t_R_ (min)	UV (nm)	[M − H]^−^	[M + H]^+^	Fragments	Standards	Quantity (mg/Kg) Extract ± SD	References
**1**	Syringic acid	9.23	280	197	-	-	-	Nq	[14]
**2**	Caffeic acid	15.31	322	179	-	-	-	Nq	[14]
**3**	Apigenin-6,8-di-C-glucoside	17.20	270, 336	593	595	-	Apigenin	1623.53 ± 288.42	[15,16]
**4**	Lithospermic acid A isomer	22.25	284, 344	537	-	339	-	Nq	[17]
**5**	Unknown	22.93	287, 331	555	-	359(+)	-	-	[18]
**6**	Luteolin glucoside	25.06	286, 336	447	449	287(+)	Kaempferol-glucoside	185.92 ± 37.27	[5,18]
**7**	Luteolin glucuronide	25.27	253, 343	461	463	287(+)	Kaempferol-glucoside	258.28 ± 50.75	[15,18]
**8**	Salvianolic acid I	34.50	309	537	493, 341	297(+)	-	Nq	[19]
**9**	Rosmarinic acid	32.55	289, 328	359	-	-	Rosmarinic acid	48128.62 ± 8077.44	[15,18,20,21]
**10**	Melitric acid B	37.03	286, 310	519	521	-	-	Nq	[18]
**11**	Melitric acid A	37.52	287, 312	537	539	-	-	Nq	[18]
**12**	Unknown	40.27	287, 327	605	607	271(+)	-	-	[20]
**13**	Diosmetin	40.50	286, 332	299	301	-	Apigenin	263.56 ± 26.02	[18]
**14**	Jaceosidin	41.76	283, 341	329	331		-	Nq	[21]
**15**	Apigenin	44.01	288, 332	269	271	-	Apigenin	89.64 ± 14.49	[15,18,20]
**16**	Cirsilineol	47.71	284, 339	-	345	-	-	Nq	[5,18,22]

**Table 3:** Determination of minimum inhibitory concentration and minimum bactericidal concentration exhibited by *Origanum compactum* ethanolic extract against bacterial strains (mg/mL).

Bacteria	MIC	MBC	MBC/MIC
*Escherichia coli*	1.30 ± 0.11	41.66 ± 0.20	32
*Salmonella typhimirium*	20.83 ± 0.20	166.66 ± 0.18	8
*Staphyloccocus aureus*	41.66 ± 0.15	166.66 ± 0.11	4
*Listeria monocytogenes*	41.66 ± 0.19	83.33 ± 0.15	2

**Table 4:** Reagents used in the search for secondary metabolites families.

Family Sought	Reagents and/or Reactions Used
Alcaloids	Dragendorff reagent and Mayer reagent
Tannins	Ferric chloride reaction and Stiasny reagent
Sterols and Triterpenes	Liebermann-Buchard reaction
Flavonoids	cyanidin reaction
Reducing compounds	Fehling’s solution
Saponosides	stirring the aqueous solution

## Data Availability

Not applicable.

## Sample Availability

Samples of the extract and the plant are available from the authors.
